# Topographic Expression of IL-1β and Inflammasome-Associated Proteins in Lesional, Perilesional, and Distant Hidradenitis Suppurativa Skin

**DOI:** 10.3390/ijms27209085

**Published:** 2026-10-13

**Authors:** Miłosz Lewandowski, Magdalena Górska-Ponikowska, Anna Jakubczyk-Słabicka, Zuzanna Świerczewska, Wioletta Barańska-Rybak

**Affiliations:** 1 Department of Dermatology, Venereology and Allergology, Faculty of Medicine, Medical University of Gdansk, Smoluchowskiego 17 Street, 80-214 Gdansk, Poland; 2 Department of Dermatology, Venereology and Allergology, University Clinical Centre in Gdansk, Dębinki 7 Street, 80-952 Gdansk, Poland; 3 Department of Medical Chemistry, Medical University of Gdansk, Debinki 1 Street, 80-211 Gdansk, Poland; 4 Department of Biophysics, Institute of Biomaterials and Biomolecular Systems, University of Stuttgart, 70569 Stuttgart, Germany; 5 Euro-Mediterranean Institute of Science and Technology, 90139 Palermo, Italy

**Keywords:** hidradenitis suppurativa, inflammasome, NLRP3, interleukin-1β, IL-18, caspase-1, Western blot, perilesional skin, inflammatory field, adalimumab

## Abstract

Hidradenitis suppurativa (HS) is a chronic inflammatory skin disease characterized by recurrent nodules, abscesses, draining tunnels, and scarring. Dysregulated IL-1 and inflammasome-associated pathways have been implicated in its pathogenesis, but their spatial distribution beyond clinically apparent lesions remains incompletely characterized. To assess topographic expression of NLRP3, caspase-1, IL-1β, and IL-18 across lesional, perilesional, and distant HS skin. Skin samples were obtained from eight biologic-untreated patients with HS, six patients receiving adalimumab, and five healthy controls. In HS, matched 6 mm full-thickness biopsies were obtained from the same axilla at an active inflammatory lesion (0 cm), clinically normal-appearing skin 2 cm from the visible lesion edge, and clinically uninvolved skin 10 cm from the lesion edge. Protein abundance was assessed by Western blot. Primary topographic effects were evaluated using mixed-design ANOVA with Benjamini–Hochberg FDR correction, supplemented by non-parametric and exploratory contrast analyses. No protein showed an FDR (false discovery rate)-significant overall site effect or cohort × site interaction in the primary analysis. In biologic-untreated HS, IL-18 showed an FDR-significant topographic effect (q = 0.0444), with higher expression at 0 cm than at 10 cm (q = 0.0469). An exploratory post hoc 2-versus-10 cm analysis identified a highly concordant IL-1β pattern: expression was higher at 2 cm in 7/8 biologic-untreated and 6/6 adalimumab-treated patients. Fisher’s combined probability remained significant after BH correction across the four proteins evaluated for this contrast (*p* = 0.00421, q4 = 0.0168), whereas comprehensive correction across all 12 protein × contrast combinations yielded q12 = 0.0505 and did not meet q < 0.05. Overall, 13/14 patients showed the same direction (92.9%; exact 95% CI 66.1–99.8%). IL-18 provided the clearest FDR-supported secondary topographic finding. IL-1β showed a highly concordant but hypothesis-generating perilesional-to-distant spatial pattern, whereas total NLRP3 and caspase-1 did not show robust FDR-adjusted spatial variation.

## 1. Introduction

Hidradenitis suppurativa (HS) is a chronic inflammatory dermatosis characterized by recurrent, painful nodules, abscesses, subcutaneous sinus tracts, and subsequent scarring. The condition is widely regarded as one of the most burdensome disorders in dermatology, owing to its chronic course, pain, and purulent discharge, which collectively are associated with a marked impairment in patients’ quality of life [1]. Epidemiological estimates vary across studies; however, most reports suggest a prevalence below 1%, with a recent meta-analysis indicating an overall prevalence of approximately 0.4% [2]. Despite extensive research efforts, the pathogenesis of hidradenitis suppurativa remains incompletely elucidated. HS is currently regarded as a heterogeneous, multifactorial inflammatory disease arising from the interplay between polygenic susceptibility, lifestyle-related factors, and aberrant activation of both innate and adaptive immune responses, together with alterations in the epithelial biology of the hair follicle unit. Earlier pathogenic models proposed primary, noninflammatory follicular occlusion as the initiating event; however, accumulating evidence indicates that infundibular hyperkeratinization and comedogenesis may also develop secondary to early, subclinical inflammatory processes detectable even in clinically non-lesional skin. Consequently, the temporal and causal relationship between follicular hyperkeratinization and inflammation remains unresolved. Within this framework, activation of innate immune pathways contributes to disease amplification but does not fully account for the immunopathology of HS, which also involves robust adaptive immune responses, including T-cell and B-cell-mediated mechanisms [3,4,5,6]. Van der Zee et al. demonstrated increased levels of TNF-α, IL-1β, and IL-10 in HS skin, supporting the rationale for targeting TNF-α and IL-1β in HS. Witte-Händel et al. subsequently showed that the IL-1 pathway is hyperactive in HS and contributes to inflammatory cell infiltration and tissue destruction. Additional studies have localized proinflammatory mediators to keratinocyte-, neutrophil-, and macrophage-rich compartments, further supporting the concept that HS inflammation is sustained by multiple interacting cellular sources. Inflammasome-related mechanisms have also been implicated in HS and other inflammatory skin diseases. Inflammasomes are cytosolic multiprotein complexes that sense microbial and danger-associated signals and promote inflammatory caspase activation, leading to maturation of IL-1β and IL-18. In skin biology, inflammasome signalling has been linked to several immune-mediated and autoinflammatory dermatoses, including psoriasis, or acneiform inflammation. In HS specifically, prior studies have reported increased epidermal inflammasome-related readouts, increased expression of P2X7R/NLRP3/IL-1β pathway components in lesional skin, enrichment of inflammasome-related genes within the myeloid compartment, and reduction of inflammatory mediator release after pharmacological NLRP3 inhibition in HS skin explants. These observations support the biological plausibility of investigating NLRP3, caspase-1, IL-1β, and IL-18 across different HS skin compartments [3,7,8,9,10,11,12,13].

In the present study, we assessed the tissue-level expression of selected inflammasome-associated proteins in HS skin. We analyzed NLRP3, caspase-1, IL-1β, and IL-18 in active lesional skin, clinically normal-appearing perilesional skin located 2 cm from the lesion, and more distant clinically uninvolved skin located 10 cm from the lesion. The novelty of this study lies in the matched topographic protein-level assessment of IL-1/inflammasome-associated mediators across lesional, perilesional, and distant HS skin, combined with comparison between biologic-untreated patients, adalimumab-treated patients, and healthy controls. This study explored whether inflammasome-associated protein expression extends beyond clinically apparent HS lesions and whether clinically normal-appearing perilesional skin represents a distinct tissue compartment rather than biologically uninvolved control skin.

## 2. Results

The present cohort comprised eight patients with hidradenitis suppurativa not receiving biologic therapy, six patients with hidradenitis suppurativa treated with adalimumab, and five healthy controls. The cohorts were broadly comparable in age, with median ages of 31.0 years in biologic-untreated HS patients, 32.5 years in adalimumab-treated HS patients, and 32.0 years in healthy controls. Males comprised 75.0% of biologic-untreated HS patients, 83.3% of adalimumab-treated HS patients, and 60.0% of healthy controls. Mean BMI was similar in both HS groups and lower in controls. DLQI was descriptively much higher in the non-biologic HS group than in the adalimumab-treated group. Hurley stage III predominated in the non-biologic cohort, whereas Hurley stage II predominated in the adalimumab-treated cohort (Table 1).

**Figure 1:**
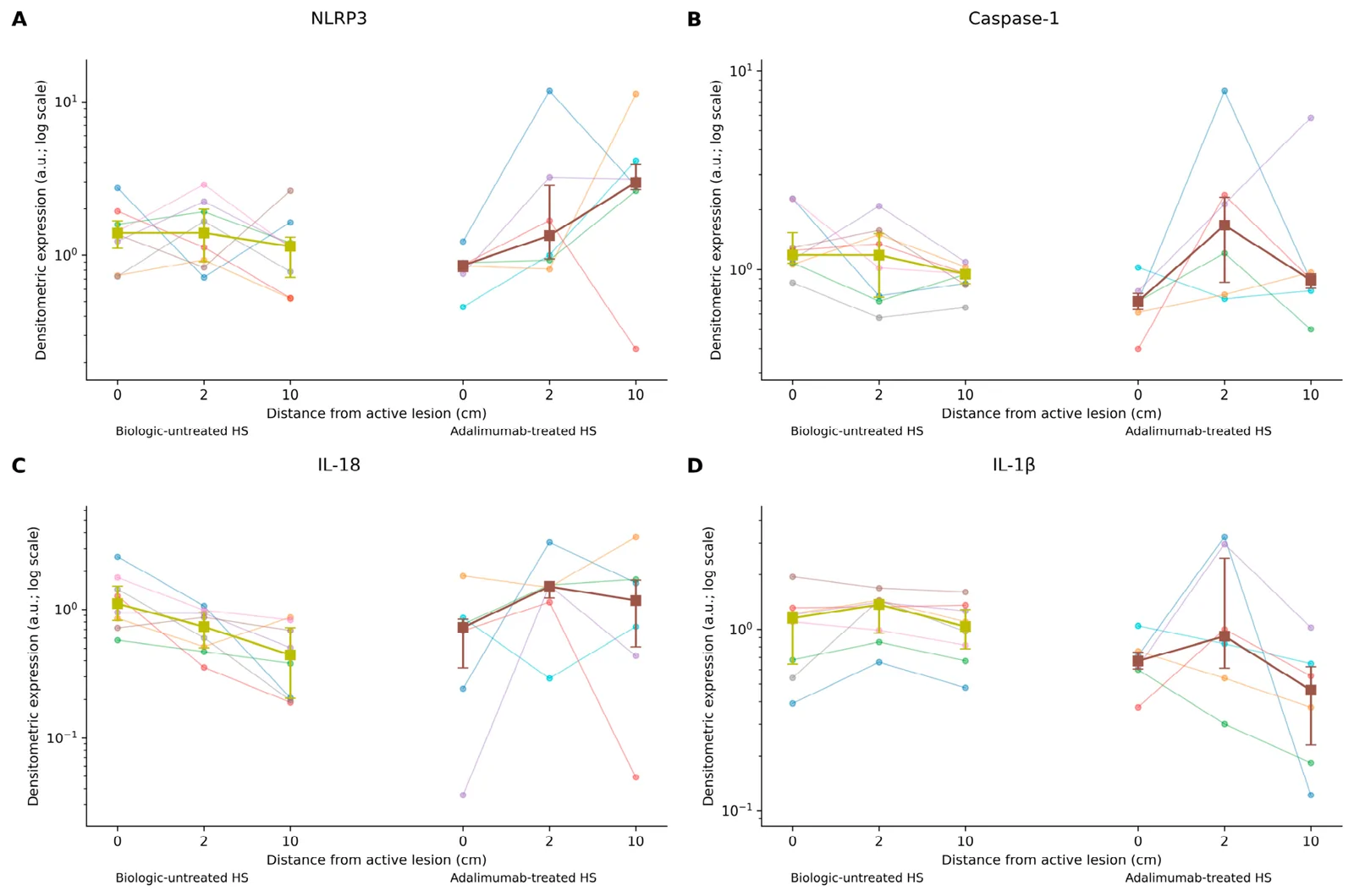
Topographic expression profiles of inflammasome-related proteins in hidradenitis suppurativa skin. Semiquantitative densitometric expression values of (**A**) NLRP3, (**B**) caspase-1, (**C**) IL-18, and (**D**) IL-1β are shown for skin sampled at 0, 2, and 10 cm from an active HS lesion in biologic-untreated and adalimumab-treated patients. The 0 cm sample represents active lesional skin, the 2 cm sample clinically normal-appearing perilesional skin, and the 10 cm sample clinically uninvolved distant skin. Individual patients are represented by connected points to visualize within-patient spatial trajectories. Squares indicate cohort medians and error bars represent the interquartile range. The *y*-axis is displayed on a logarithmic scale because of the right-skewed distribution of several protein-expression measurements. Expression values are presented in arbitrary units (a.u.) and were analyzed without normalization to β-actin. Corresponding mixed-design ANOVA results for the site effect and cohort × site interaction are reported in Table 2A,B.

In the primary mixed-design ANOVA of log2-transformed expression values, none of the four proteins showed an overall site main effect or cohort × site interaction that remained statistically significant after Benjamini–Hochberg false-discovery-rate correction (Table 2A,B; Figure 1). ANOVA results are reported as F(df1, df2), where df1 and df2 denote the Greenhouse–Geisser-adjusted numerator and denominator degrees of freedom, respectively. IL-1β showed a nominal site main effect (F(1.894, 22.728) = 4.409, *p* = 0.0257, q = 0.1029, partial η^2^ = 0.269). Nominal cohort × site interactions were observed for caspase-1 (F(1.833, 21.997) = 4.327, *p* = 0.0287, q = 0.0940, partial η^2^ = 0.265) and IL-18 (F(1.886, 22.628) = 3.577, *p* = 0.0470, q = 0.0940, partial η^2^ = 0.230), but neither remained significant after FDR correction. NLRP3 showed neither a significant site main effect nor a significant cohort × site interaction.

In cohort-specific non-parametric sensitivity analyses, IL-18 was the only protein showing an FDR-significant overall topographic effect, observed in biologic-untreated HS (Friedman χ^2^ = 9.00, *p* = 0.0111, q = 0.0444; Kendall’s W = 0.563) (Table 3). Post hoc exact Wilcoxon testing showed higher IL-18 expression at 0 cm than at 10 cm (median 0/10 ratio = 2.01; Hodges–Lehmann ratio = 2.72, 95% CI 1.22–7.29; *p* = 0.0156, q = 0.0469), whereas the 0-vs.-2 cm and 2-vs.-10 cm comparisons did not remain significant after correction. No other cohort-specific Friedman analysis reached q < 0.05; pairwise results arising from these non-significant omnibus analyses were therefore retained as exploratory findings in Supplementary Table S1.

**Figure 2:**
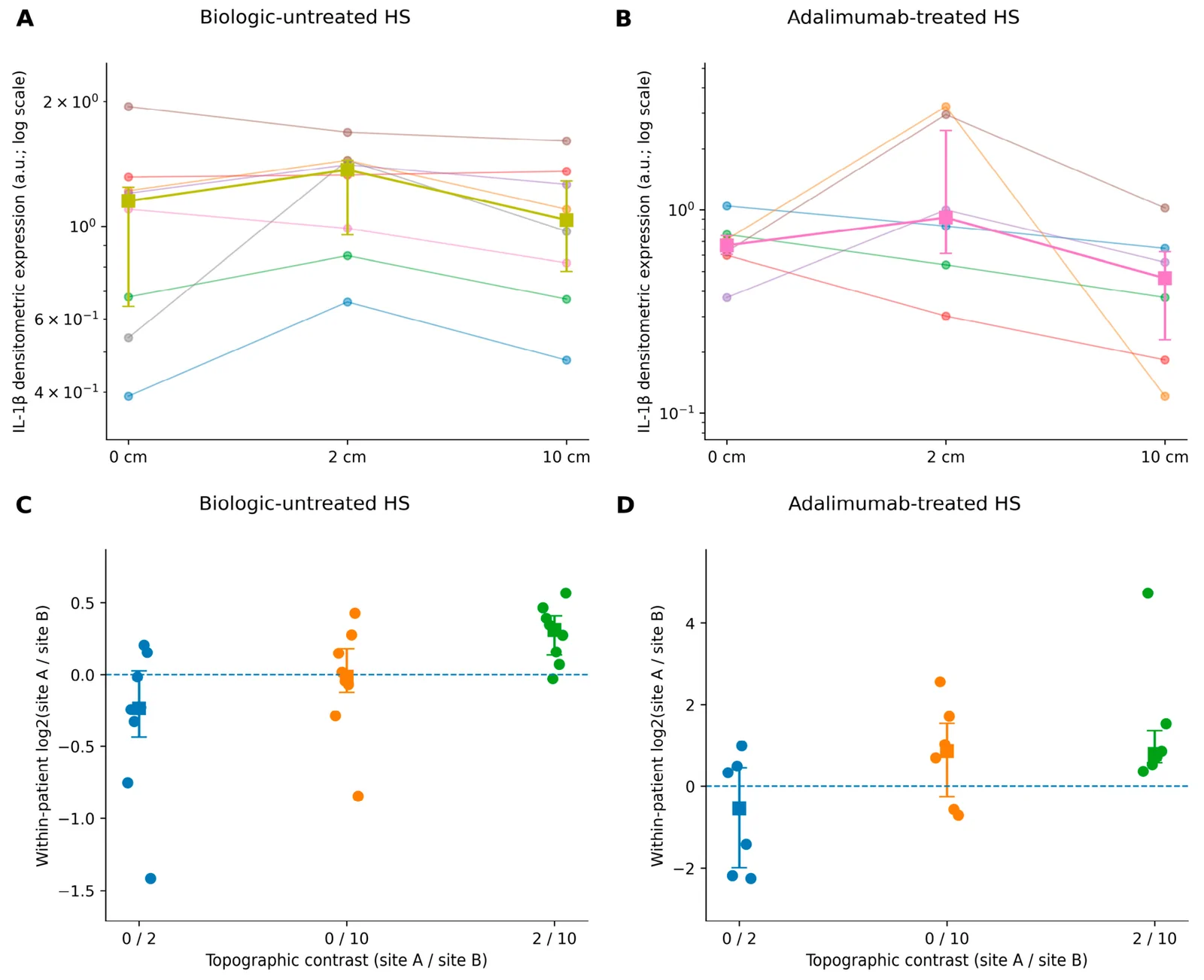
Within-patient topographic distribution of IL-1β expression in hidradenitis suppurativa skin. (**A**,**B**) Individual IL-1β expression trajectories across lesional skin at 0 cm, clinically normal-appearing perilesional skin at 2 cm, and clinically uninvolved distant skin at 10 cm in (**A**) biologic-untreated and (**B**) adalimumab-treated patients. Individual patients are represented by connected points; squares indicate cohort medians and error bars represent the interquartile range. Expression values are shown in arbitrary units (a.u.) on a logarithmic scale. (**C**,**D**) Patient-level within-subject log2 expression ratios for all three possible spatial contrasts (0/2 cm, 0/10 cm, and 2/10 cm) in (**C**) biologic-untreated and (**D**) adalimumab-treated HS. Each point represents one patient. Squares indicate median log2 ratios and error bars represent the interquartile range. The horizontal dashed line at 0 represents equal expression at the two compared sites; positive values indicate higher expression at the numerator site, whereas negative values indicate higher expression at the denominator site. The most consistent directional pattern was observed for the 2 cm versus 10 cm contrast, with higher IL-1β expression at 2 cm in 7/8 biologic-untreated patients and 6/6 adalimumab-treated patients. This contrast was not prespecified and is interpreted as exploratory and hypothesis-generating. Exact inferential statistics, including q4 and the comprehensive q12 correction, are reported in Table 4 and Supplementary Tables S1 and S2.

For IL-1β, post hoc evaluation of all three paired spatial contrasts identified the 2 cm versus 10 cm comparison as the most consistent cross-cohort pattern (Figure 2; Table 4). Because this contrast was not prespecified before examination of the data, the following analyses are considered exploratory and hypothesis-generating. IL-1β expression was higher at 2 cm in 7 of 8 biologic-untreated patients and in all 6 adalimumab-treated patients. Cohort-specific exact Wilcoxon tests yielded *p* = 0.0156 in biologic-untreated HS (median 2/10 ratio = 1.24; Hodges–Lehmann ratio = 1.21, 95% CI 1.05–1.38; q4 = 0.0625) and *p* = 0.0312 in adalimumab-treated HS (median ratio = 1.73; Hodges–Lehmann ratio = 1.93, 95% CI 1.29–26.65; q4 = 0.1250). When the two independent cohort-specific *p*-values were combined using Fisher’s method, the result remained significant after Benjamini–Hochberg correction across the four proteins evaluated for this 2-vs.-10 cm contrast (*p* = 0.00421, q4 = 0.0168). However, the comprehensive correction across all 12 protein × contrast combinations yielded q12 = 0.0505 and therefore did not meet the q < 0.05 FDR threshold. A pooled exact paired Wilcoxon analysis, retained only as a supportive sensitivity analysis, yielded *p* = 0.000244, with 13 of 14 patients (92.9%; exact 95% CI 66.1–99.8%) showing the same direction of effect. The magnitude of the 2 cm/10 cm contrast differed between treatment-status cohorts (*p* = 0.00799, q4 = 0.0320), but this between-cohort difference was not interpreted as a treatment effect because treatment status was confounded with baseline disease severity. The 0-vs.-2 cm and 0-vs.-10 cm IL-1β contrasts did not show a consistent statistically supported difference across cohorts.

No statistical evidence of a systematic between-gel effect was detected after adjustment for treatment-status cohort (all BH-adjusted q ≥ 0.6961; Supplementary Table S3). Thus, gel identity did not significantly account for additional between-patient variation in mean log2 expression in the present dataset, although smaller batch effects cannot be excluded. Secondary comparisons with healthy controls likewise yielded no FDR-significant differences after multiplicity correction (Supplementary Table S4).

## 3. Discussion

Hidradenitis suppurativa is increasingly understood as a heterogeneous inflammatory disorder in which follicular disruption, innate and adaptive immune dysregulation, microbial and danger-associated signals, and abnormal tissue remodelling interact to sustain chronic inflammation [3,5,6,14]. Within this network, the IL-1 axis has consistently emerged as an important component of HS biology [3,15,16]. In the present study, however, the primary mixed-design analysis did not identify an FDR-significant overall site effect or cohort × site interaction for any investigated protein. The most conventional multiplicity-adjusted topographic finding was instead observed for IL-18 in biologic-untreated HS, where the Friedman omnibus test and the subsequent 0-versus-10 cm post hoc comparison both remained significant after FDR correction. IL-1β showed a different pattern. The 2-versus-10 cm contrast was not prespecified before examination of the data and is therefore considered exploratory and hypothesis-generating. Nevertheless, the direction was highly concordant across treatment-status cohorts: IL-1β was higher at 2 cm in 7 of 8 biologic-untreated patients and all 6 adalimumab-treated patients. Fisher’s combined probability remained significant after Benjamini–Hochberg correction across the four proteins evaluated for this selected contrast (q4 = 0.0168), whereas the comprehensive correction across all 12 protein × contrast combinations yielded q12 = 0.0505 and did not meet the predefined FDR threshold. The IL-1β result should therefore be interpreted as a strong exploratory spatial signal rather than a confirmatory finding.

The IL-1β pattern is biologically consistent with evidence that clinically normal-appearing skin adjacent to HS lesions is not necessarily immunologically uninvolved. Navrazhina et al. used a directly comparable topographic framework and showed that perilesional skin sampled 2 cm from an inflammatory nodule clustered with lesional rather than non-lesional skin and displayed enrichment of inflammatory and neutrophil-associated pathways [17]. These observations provide an independent molecular context for the present 2-versus-10 cm signal. At the same time, the absence of a statistically significant 0-versus-2 cm IL-1β difference in our cohort does not establish biological equivalence between lesional and perilesional skin. The most defensible interpretation is that the 2 cm compartment may retain inflammatory abnormalities relative to more distant clinically uninvolved skin. This is consistent with proposed standardized distinctions between lesional, perilesional, and uninvolved HS skin [18]. Perilesional skin has also been shown to capture molecular treatment-associated changes in an interventional brodalumab study, supporting its relevance as a distinct translational sampling compartment [19].

IL-18 provides complementary evidence that spatial inflammatory biology in HS is not uniform across analytes. In biologic-untreated HS, IL-18 was higher at the lesional 0 cm site than at the 10 cm site, with both the omnibus and post hoc analyses remaining significant after FDR correction. This pattern was not reproduced in the adalimumab-treated cohort and should therefore be considered cohort-restricted. Previous studies have demonstrated dysregulation of the IL-1 pathway in HS, including increased IL-1β and broad IL-1-associated inflammatory activity [15,16]. Our results extend this literature by examining matched spatial compartments within the same patient, but they do not demonstrate a generalized increase in absolute cytokine abundance relative to healthy skin, because no comparison with healthy controls remained significant after multiplicity correction.

In contrast, total NLRP3 and caspase-1 abundance did not show robust FDR-adjusted topographic differences. This does not exclude functional involvement of inflammasome pathways in HS. Prior work has demonstrated inflammasome-related activity in HS tissue, increased P2X7R/NLRP3/IL-1β-associated immunoreactivity, and reduction of inflammatory mediator release after pharmacological NLRP3 inhibition [9,10,11]. These studies interrogated cellular localization, transcriptional programmes, activated proteins, or functional secretion, whereas the present study measured semiquantitative total protein abundance in bulk-tissue lysates. Total protein abundance is not equivalent to inflammasome assembly or enzymatic activation, and IL-1β can also undergo inflammasome-independent proteolytic processing [20,21]. Importantly, bulk-tissue differences may additionally reflect differences in cellular and tissue composition rather than altered protein expression within individual cells. Active lesional, perilesional, and distant skin may differ in the relative proportions of inflammatory cells, keratinocytes, adnexal structures, and stromal components. Macrophage- and neutrophil-rich inflammation is well described in HS [17,22]. Because cellular composition was not directly quantified here, the present data cannot determine whether the observed cytokine patterns reflect altered expression per cell, differences in tissue composition, functional inflammasome activation, alternative IL-1 processing, or a combination of these mechanisms.

The observed difference in the magnitude of the exploratory IL-1β 2-versus-10 cm contrast between treatment-status cohorts also requires restraint. Hurley stage III predominated in the biologic-untreated cohort, whereas Hurley stage II predominated in the adalimumab-treated cohort. Baseline disease severity was therefore confounded with treatment status, in addition to the non-randomized cross-sectional design. Moreover, the global IL-1β cohort × site interaction was not FDR-significant. Between-cohort differences in contrast magnitude consequently cannot be attributed to a pharmacodynamic effect of adalimumab and are best regarded as evidence of heterogeneity requiring longitudinal within-patient confirmation.

The findings remain relevant to the broader IL-1 pathway, for which clinical studies of anakinra, bermekimab, lutikizumab, and canakinumab have provided variable levels of therapeutic evidence [23,24,25,26]. However, the present molecular data do not identify an optimal therapeutic target or predict response to IL-1-directed treatment. Their main translational implication is methodological: clinically normal-appearing perilesional skin should not automatically be assumed to represent biologically uninvolved internal control tissue, and spatial sampling may reveal inflammatory patterns that are obscured by conventional lesion-versus-control comparisons.

Taken together, these data support a spatially heterogeneous and protein-specific model of IL-1-associated inflammation in HS. IL-18 provided the clearest FDR-supported secondary topographic result, whereas IL-1β showed a highly concordant but post hoc 2-versus-10 cm pattern that remained significant within the four-protein exploratory contrast family but not under the comprehensive 12-test FDR correction. Total NLRP3 and caspase-1 did not show parallel spatial gradients. These findings warrant validation in larger, prospectively characterized cohorts using standardized spatial sampling together with cell-resolved and functional assays.

## 4. Materials and Methods

Adult patients with hidradenitis suppurativa were prospectively enrolled after providing voluntary written informed consent. The study population included patients aged ≥18 years with a dermatologist-confirmed diagnosis of HS assessed using the Hurley staging system. Hurley stage I denotes inflammatory nodules or abscesses without sinus tracts or scarring; stage II recurrent abscesses with sinus tract formation and scarring separated by clinically unaffected skin; and stage III diffuse or near-diffuse involvement of an anatomical region with multiple interconnected sinus tracts and abscesses. Although the study protocol allowed inclusion of Hurley stage I–III disease, all enrolled patients had Hurley stage II or III disease. In patients with HS, all biopsies were obtained from axillary skin. For each patient, the three matched samples were taken from the same left or right axilla using 6 mm full-thickness punch biopsies. The 0 cm sample was obtained from a clinically visible active inflammatory HS lesion; lesion selection was based on visible inflammatory activity and was not restricted to a specific morphological lesion subtype. The 2 cm sample was obtained from clinically normal-appearing perilesional skin and the 10 cm sample from clinically uninvolved distant skin. Both distances were measured from the clinically visible edge of the active inflammatory lesion. Healthy control skin samples were obtained from adults without a history or clinical evidence of HS or other active inflammatory skin disease. The study groups were defined as biologic-untreated HS, adalimumab-treated HS, and healthy controls. Exclusion criteria included age <18 years, pregnancy or lactation, inability or unwillingness to provide written informed consent, active systemic infection, clinically relevant immunodeficiency, concomitant inflammatory or autoimmune skin disease that could interfere with tissue-level inflammatory analyses, and use of systemic immunomodulatory or immunosuppressive treatment not compatible with the predefined study group allocation. The biologic-untreated HS group included patients not receiving biologic therapy at the time of sampling, whereas the adalimumab-treated HS group included patients receiving adalimumab. Treatment status and clinical disease severity were recorded at the time of biopsy. The study was conducted in accordance with the Declaration of Helsinki and was approved by the Independent Bioethics Committee for Scientific Research at the Medical University of Gdańsk (KB/307/2023).

### 4.1. Sample Preparation Protocol

Before homogenization, samples from patients with hidradenitis suppurativa had been weighed and later divided into smaller fragments using a surgical scalpel to enhance tissue homogenization.

After sample preparation, the specimens were homogenized with a manual tissue homogenizer and an IKA Dispersers T 10 basic ultra-turrax mechanical homogenizer, specifically engineered for homogenizing soft tissue within Eppendorf tubes. The homogenization procedure was conducted at a lowered temperature with the incorporation of a lysis buffer (0 to 4 °C). After homogenization, samples were subjected to the Bradford assay. Thirty microliters of supernatant were removed from the homogenate sample and 1.5 mL of Bradford reagent was added. The samples were then incubated for approximately 5 min at room temperature, and absorbance was measured. Bovine serum albumin (BSA) standard solutions ranging from 6.25 to 200 µg/mL were used to generate the calibration curve. Absorbance was measured at 595 nm. The standard curve was applied to determine total protein concentration in tissue homogenates prior to Western blot analysis.

### 4.2. Western Blot Analysis

Western blotting was performed as previously described [27]. Briefly, 22.5 μg of total protein per sample, determined using the Bradford assay, was subjected to polyacrylamide gel electrophoresis using Mini-PROTEAN TGX Precast Gels (Bio-Rad Laboratories, Hercules, CA, USA) and transferred using the Trans-Blot Turbo Transfer System (Bio-Rad Laboratories, Hercules, CA, USA). Membranes were incubated overnight at 4 °C with primary antibodies against caspase-1 (#3866, D7F10), IL-1β (#12703, D3U3E), IL-18 (#67775, E3G8R), and NLRP3 (#15101, D4D8T) (Cell Signaling Technology, Danvers, MA, USA; 1:1000). β-actin was used during experimental quality control as a loading-control marker (1:50,000); however, quantitative lane-level β-actin densitometry suitable for retrospective normalization was not available for the present analysis. Chemiluminescent signals were acquired using an ImageQuant LAS 500 system (GE Healthcare Life Sciences, Uppsala, Sweden). For quantitative analyses, target-protein densitometric values were therefore analyzed directly as semiquantitative arbitrary units and were not divided by β-actin or rescaled to healthy-control values. Equal total-protein input was established before electrophoresis, and all three matched biopsy samples from an individual HS patient were analyzed on the same gel. These measures reduce, but do not eliminate, susceptibility to technical loading, transfer, or detection variability. The resulting values should therefore be interpreted as relative semiquantitative measures of protein abundance rather than absolute protein concentrations. Uncropped Western blot images for all analyzed target proteins are provided in the Supplementary Materials.

### 4.3. Statistical Analysis

Quantitative Western blot data for NLRP3, caspase-1, IL-18, and IL-1β were analyzed as semiquantitative densitometric expression values expressed in arbitrary units, without normalization to β-actin and without rescaling healthy-control measurements to a reference value. Because several analytes showed positively skewed distributions and occasional high-intensity observations, expression values were log2-transformed for the primary parametric analyses. Topographic variation within HS skin was evaluated using a two-factor mixed-design analysis of variance (ANOVA), with (1) biopsy site (0, 2, and 10 cm from the active lesion) as the within-subject factor and (2) treatment-status cohort (biologic-untreated versus adalimumab-treated) as the between-subject factor. The primary terms of interest were the site main effect, assessing overall spatial variation in protein expression, and the cohort × site interaction, assessing whether the spatial expression profile differed between treatment-status cohorts. The cohort main effect was included in the model but was not considered a primary inferential endpoint. Greenhouse–Geisser correction was applied to within-subject effects to account for potential departures from sphericity, and partial eta-squared (partial η^2^) was reported as an effect-size measure. Benjamini–Hochberg false-discovery-rate (FDR) correction was applied separately across the four proteins for the site-effect and cohort × site interaction families.

Given the limited cohort sizes and the non-Gaussian distribution of several measurements, cohort-specific non-parametric analyses were additionally performed as sensitivity analyses. Within each treatment-status cohort, overall differences among the three matched biopsy sites were assessed using the Friedman test, with Kendall’s W reported as an effect-size measure. Benjamini–Hochberg correction was applied across the four proteins within each cohort. To provide a complete characterization of the spatial expression pattern, all three possible paired site contrasts (0 versus 2 cm, 0 versus 10 cm, and 2 versus 10 cm) were calculated for each protein using exact Wilcoxon signed-rank tests. Formal post hoc interpretation was restricted to comparisons supported by the corresponding false-discovery-rate-adjusted omnibus analysis, with multiplicity correction applied across the three pairwise contrasts; pairwise results obtained in the absence of an FDR-significant omnibus effect were retained for transparency but considered exploratory.

Because the 2 cm versus 10 cm pattern emerged during examination of the data and was not prespecified in an a priori statistical analysis plan, this comparison was treated as an exploratory, hypothesis-generating contrast. The 2 cm versus 10 cm contrast was evaluated separately within each treatment-status cohort using exact paired Wilcoxon signed-rank tests, with within-patient expression ratios and rank-biserial correlations used to characterize effect magnitude and direction. The same spatial contrast was evaluated across all four proteins; Benjamini–Hochberg correction across these four protein-specific tests is denoted q4. Because the contrast itself was selected post hoc from the three possible pairwise spatial comparisons, a more comprehensive Benjamini–Hochberg correction across all 12 protein × contrast combinations was additionally calculated and is denoted q12. Cohort-specific *p*-values were combined using Fisher’s probability method without pooling patient-level measurements, and the direction of cohort-specific effects was examined alongside the combined result. Between-cohort heterogeneity in contrast magnitude was assessed using an exact Mann–Whitney test applied to patient-level log2 expression ratios. A pooled paired analysis of all HS patients and an exact binomial sign test were retained only as supportive sensitivity analyses. For key paired contrasts retained for interpretation, Hodges–Lehmann estimates with exact 95% confidence intervals based on the paired signed-rank distribution were calculated on the log2 difference scale and back-transformed to expression ratios. For the pooled directional consistency of the IL-1β 2 cm versus 10 cm contrast, an exact Clopper–Pearson 95% confidence interval was calculated. Confidence intervals were descriptive and were not adjusted for multiplicity.

Because all three biopsy samples from a given HS patient were analyzed on the same Western blot gel, within-patient topographic comparisons were less susceptible to uniform whole-gel shifts than comparisons between independent subjects, particularly when expressed as within-patient log2 ratios. Potential between-gel variability was nevertheless evaluated separately. For each protein, the patient-level mean log2 expression across the three biopsy sites was analyzed using a linear model including treatment-status cohort and gel as categorical predictors. The overall contribution of gel was evaluated using a partial F test comparing the full model containing cohort and gel with a reduced model containing cohort alone. Additional exact permutation-based between-gel comparisons were performed within treatment-status strata represented on more than one gel. Benjamini–Hochberg correction was applied across the four proteins for gel-level analyses. Because treatment-status cohort and gel allocation were not fully crossed, these analyses were regarded as sensitivity analyses rather than definitive tests excluding batch effects.

Healthy controls contributed a single skin sample and were therefore not included in the repeated-measures topographic analyses. For secondary comparisons with healthy skin, control values were compared with biologic-untreated and adalimumab-treated HS values separately at each HS biopsy site using the Kruskal–Wallis test. Benjamini–Hochberg correction was applied across the 12 protein × site comparisons. Analyses restricted to samples measured on the same gel were additionally performed as sensitivity analyses where the study design permitted such comparisons.

All statistical tests were two-sided. Raw *p*-values are reported together with Benjamini–Hochberg-adjusted *p*-values (q-values), with q < 0.05 considered statistically significant within the specified multiplicity family. For the exploratory IL-1β 2 cm versus 10 cm analysis, q4 and q12 are reported together so that significance within the four-protein contrast family is distinguished from the comprehensive correction across all protein × contrast combinations. Statistical interpretation incorporated the primary mixed-design analysis, effect size, confidence intervals, direction and consistency across subjects, and multiplicity-adjusted sensitivity analyses rather than unadjusted *p*-values alone. Analyses were performed using Python 3.13.5 with NumPy 2.3.5 and SciPy 1.17.0.

## 5. Conclusions

The present study supports spatial heterogeneity in IL-1-family cytokine expression within HS skin. IL-18 provided the clearest FDR-supported secondary topographic finding, with higher expression in lesional than distant skin in the biologic-untreated cohort. IL-1β showed a highly concordant exploratory pattern between clinically normal-appearing perilesional skin at 2 cm and more distant clinically uninvolved skin at 10 cm. Fisher’s combined result remained significant after FDR correction across the four proteins evaluated for this selected contrast (q4 = 0.0168), but the comprehensive 12-test correction yielded q12 = 0.0505 and did not meet the q < 0.05 threshold; the IL-1β finding should therefore be regarded as hypothesis-generating rather than confirmatory. Total NLRP3 and caspase-1 abundance did not demonstrate robust FDR-adjusted spatial differences.

These findings suggest that clinically normal-appearing perilesional HS skin may retain inflammatory abnormalities relative to more distant skin, but they do not establish functional inflammasome activation, altered expression per cell, or treatment-related effects. Validation in larger, clinically balanced, longitudinal cohorts using cell-resolved and functional approaches is required.

### 5.1. Strengths and Limitations

A major strength of this study is the paired topographic design, with lesional, 2 cm perilesional, and 10 cm distant skin obtained from the same patient and analyzed on the same Western blot gel. This approach reduces interindividual variability and makes within-patient spatial contrasts less susceptible to uniform whole-gel shifts.

The principal limitations are the modest sample size and the cross-sectional, non-randomized comparison of treatment-status cohorts. Hurley stage III predominated in the biologic-untreated cohort, whereas Hurley stage II predominated in the adalimumab-treated cohort; baseline disease severity was therefore confounded with treatment status, precluding attribution of between-cohort differences to a pharmacodynamic effect of adalimumab. Active lesions were selected on the basis of visible clinical inflammation without restriction to a specific lesion morphology, which may also contribute to biological heterogeneity. Western blotting quantified semiquantitative total protein abundance in bulk-tissue lysates and therefore cannot distinguish altered expression within individual cells from differences in cellular or tissue composition across biopsy sites. Target-protein values were analyzed without β-actin normalization because quantitative lane-level β-actin densitometry suitable for retrospective normalization was not available. Equal total-protein input was established by Bradford assay, and matched samples from each patient were analyzed on the same gel; nevertheless, residual lane-specific loading, transfer, and detection variability cannot be excluded. These limitations, together with the exploratory status of the IL-1β 2-versus-10 cm analysis, require validation in larger independent cohorts.

### 5.2. Future Directions

Future studies should validate the observed spatial patterns in larger, prospectively characterized cohorts using standardized lesion definitions and matched sampling within the same anatomical region. Longitudinal within-patient sampling before and during treatment would be particularly valuable for separating disease-severity effects from treatment-associated changes.

Mechanistic studies should complement bulk-tissue protein measurements with spatial and functional approaches, including immunohistochemistry or immunofluorescence, single-cell or spatial transcriptomics, and direct markers of inflammasome activation such as cleaved caspase-1, mature IL-1β and IL-18, ASC speck formation, and gasdermin D cleavage. Such approaches may determine whether perilesional cytokine signals reflect specific cellular sources, altered tissue composition, or functional inflammatory endotypes.

## Figures and Tables

**Table 1:** Clinical characteristics of the study groups. Abbreviations: BMI, body mass index; DLQI, Dermatology Life Quality Index; HS, hidradenitis suppurativa; IQR, interquartile range; SD, standard deviation.

Group	*n*	BMI	DLQI	Hurley Stage
HS, not biologically treated	8	Mean ± SD: 28.31 ± 3.54 Median [IQR]: 28.23 [26.44–30.82] Range: 23.29–33.56	Mean ± SD: 15.50 ± 1.93 Median [IQR]: 15.50 [14.75–17.00] Range: 12–18	Hurley II: 2/8 (25.00%) Hurley III: 6/8 (75.00%)
HS, treated with adalimumab	6	Mean ± SD: 28.51 ± 2.25 Median [IQR]: 28.88 [28.08–29.53] Range: 24.54–31.23	Mean ± SD: 7.50 ± 2.43 Median [IQR]: 7.00 [6.25–7.75] Range: 5–12	Hurley II: 4/6 (66.67%) Hurley III: 2/6 (33.33%)
Healthy controls	5	Mean ± SD: 24.03 ± 1.98 Median [IQR]: 23.45 [22.98–24.61] Range: 21.97–27.14	Not applicable	Not applicable

**Table 2:** (**A**) Primary mixed-design ANOVA: site main effect. The site main effect tests overall spatial variation across the 0-, 2-, and 10 cm biopsy sites. F denotes the ANOVA F statistic. df1 and df2 are the numerator and denominator degrees of freedom, respectively; because these are within-subject effects, the degrees of freedom were adjusted using the Greenhouse–Geisser correction and may therefore be non-integer. BH q-values are Benjamini–Hochberg-adjusted *p*-values across the four proteins. Partial η^2^ (partial eta-squared) is an effect-size measure for the tested effect relative to that effect plus its associated error variance. (**B**) Primary mixed-design ANOVA: cohort × site interaction. The cohort × site interaction tests whether the spatial 0–2–10 cm expression profile differs between biologic-untreated and adalimumab-treated HS. F denotes the ANOVA F statistic. df1 and df2 are the Greenhouse–Geisser-adjusted numerator and denominator degrees of freedom, respectively. BH q-values are adjusted across the four proteins. Partial η^2^ quantifies the magnitude of the interaction relative to the interaction plus its associated error variance.

(**A**)						
**Protein**	**F Statistic**	**df1**	**df2**	***p***	**BH q**	**Partial η^2^**
NLRP3	1.144	1.539	18.472	0.3263	0.3263	0.087
Caspase-1	1.480	1.833	21.997	0.2490	0.3263	0.110
IL-18	1.289	1.886	22.628	0.2934	0.3263	0.097
IL-1β	4.409	1.894	22.728	0.0257	0.1029	0.269
(**B**)						
**Protein**	**F Statistic**	**df1**	**df2**	***p***	**BH q**	**Partial η^2^**
NLRP3	3.106	1.539	18.472	0.0795	0.1046	0.206
Caspase-1	4.327	1.833	21.997	0.0287	0.0940	0.265
IL-18	3.577	1.886	22.628	0.0470	0.0940	0.230
IL-1β	2.526	1.894	22.728	0.1046	0.1046	0.174

**Table 3:** Cohort-specific Friedman sensitivity analyses. Overall differences across matched 0-, 2-, and 10 cm sites were assessed separately within each treatment-status cohort. BH q-values are adjusted across the four proteins within each cohort. Kendall’s W is the effect-size measure for the Friedman test; larger values indicate a stronger and more consistent within-subject topographic effect.

Protein	Cohort	*n*	Friedman χ^2^	Kendall W	*p*	BH q
NLRP3	Biologic-untreated	8	3.250	0.203	0.1969	0.1969
Caspase-1	Biologic-untreated	8	5.250	0.328	0.0724	0.1240
IL-18	Biologic-untreated	8	9.000	0.563	0.0111	0.0444
IL-1β	Biologic-untreated	8	4.750	0.297	0.0930	0.1240
NLRP3	Adalimumab-treated	6	4.000	0.333	0.1353	0.2707
Caspase-1	Adalimumab-treated	6	2.333	0.194	0.3114	0.4152
IL-18	Adalimumab-treated	6	1.000	0.083	0.6065	0.6065
IL-1β	Adalimumab-treated	6	4.333	0.361	0.1146	0.2707

**Table 4:** Selected topographic contrasts. For the IL-18 post hoc analysis, q reflects correction across the three pairwise contrasts after an FDR-significant Friedman omnibus test. The IL-1β 2-vs.-10 cm contrast was not prespecified and is interpreted as exploratory and hypothesis-generating. For these IL-1β analyses, q4 denotes Benjamini–Hochberg correction across the four proteins evaluated for the selected 2-vs.-10 cm contrast. The comprehensive correction across all 12 protein × contrast combinations yielded q12 = 0.0505 for the Fisher-combined IL-1β result and is reported in Supplementary Table S2. Hodges–Lehmann ratio estimates with 95% confidence intervals are reported in the Section 2.

Protein	Analysis Set	Contrast	Test	Effect/Direction	*p*	BH q
IL-18	Biologic-untreated	0 vs. 10 cm	Exact paired Wilcoxon	Median ratio 2.01	0.0156	0.0469
IL-1β	Biologic-untreated	2 vs. 10 cm	Exact paired Wilcoxon	7/8; median ratio 1.24	0.0156	0.0625
IL-1β	Adalimumab-treated	2 vs. 10 cm	Exact paired Wilcoxon	6/6; median ratio 1.73	0.0312	0.1250
IL-1β	Both cohorts	2 vs. 10 cm	Fisher-combined probability	Same direction; q12 = 0.0505	0.00421	0.0168
IL-1β	Pooled *n* = 14	2 vs. 10 cm	Exact paired Wilcoxon	13/14 (95% CI 66.1–99.8%); median 1.35	0.000244	0.000977
IL-1β	Between cohorts	2 vs. 10 cm	Exact Mann–Whitney	Contrast magnitude differs	0.00799	0.0320

## Data Availability

The data that support the findings of this study are available from the corresponding author upon reasonable request, subject to applicable ethical, institutional, and data-protection restrictions.

## Supplementary Materials

The following supporting information can be downloaded at https://www.mdpi.com/article/10.3390/ijms27209085/s1.
