# Supplementary material for: Topographic Expression of IL-1β and Inflammasome-Associated Proteins in Lesional, Perilesional, and Distant Hidradenitis Suppurativa Skin
Source: Int J Mol Sci. 2026 Oct 13;27(20):9085. doi: 10.3390/ijms27209085 (PMC13705355; doi:10.3390/ijms27209085)
Supplement: Supplementary file 1 [file ijms-27-09085-s001.zip › ijms-4557229-supplementary.pdf]

Table S1A. Complete pairwise topographic comparisons in the biologic-untreated cohort.

All three paired spatial contrasts were calculated for all four proteins using exact Wilcoxon signed-rank tests. q3 denotes Benjamini-Hochberg correction across the three pairwise contrasts for a given protein and cohort. Omnibus BH q is the corresponding Friedman result. Pairwise results with a non-FDR-significant omnibus test are retained for transparency and should be interpreted as exploratory.

Protein	Contrast	n	A>B	Median ratio A/B	Rank-biserial r	p	BH q3	Omnibus BH q
NLRP3	0 vs 2 cm	8	3/8	0.8104	-0.1667	0.7422	0.7422	0.1969
NLRP3	0 vs 10 cm	8	6/8	1.2951	0.5556	0.1953	0.5859	0.1969
NLRP3	2 vs 10 cm	8	6/8	1.8477	0.2778	0.5469	0.7422	0.1969
Caspase-1	0 vs 2 cm	8	4/8	1.2136	0.2222	0.6406	0.6406	0.1240
Caspase-1	0 vs 10 cm	8	8/8	1.3190	1	0.00781	0.0234	0.1240
Caspase-1	2 vs 10 cm	8	5/8	1.2392	0.5000	0.2500	0.3750	0.1240
IL-18	0 vs 2 cm	8	7/8	1.7260	0.8333	0.0391	0.0586	0.0444
IL-18	0 vs 10 cm	8	7/8	2.0087	0.9444	0.0156	0.0469	0.0444
IL-18	2 vs 10 cm	8	7/8	1.5583	0.7222	0.0781	0.0781	0.0444
IL-1β	0 vs 2 cm	8	2/8	0.8491	-0.5556	0.1953	0.2930	0.1240
IL-1β	0 vs 10 cm	8	4/8	0.9903	0.0556	0.9453	0.9453	0.1240
IL-1β	2 vs 10 cm	8	7/8	1.2395	0.9444	0.0156	0.0469	0.1240

Table S1B. Complete pairwise topographic comparisons in the adalimumab-treated cohort.

All three paired spatial contrasts were calculated for all four proteins using exact Wilcoxon signed-rank tests. q3 denotes Benjamini-Hochberg correction across the three pairwise contrasts for a given protein and cohort. Omnibus BH q is the corresponding Friedman result. Pairwise results with a non-FDR-significant omnibus test are retained for transparency and should be interpreted as exploratory.

Protein	Contrast	n	A>B	Median ratio A/B	Rank-biserial r	p	BH q3	Omnibus BH q
NLRP3	0 vs 2 cm	6	1/6	0.4884	-0.8095	0.0938	0.1406	0.2707
NLRP3	0 vs 10 cm	6	1/6	0.2927	-0.9048	0.0625	0.1406	0.2707
NLRP3	2 vs 10 cm	6	3/6	0.6929	-0.2381	0.6875	0.6875	0.2707
Caspase-1	0 vs 2 cm	6	1/6	0.4695	-0.8095	0.0938	0.2812	0.4152
Caspase-1	0 vs 10 cm	6	2/6	0.7098	-0.5238	0.3125	0.4688	0.4152
Caspase-1	2 vs 10 cm	6	3/6	1.6619	0.2381	0.6875	0.6875	0.4152
IL-18	0 vs 2 cm	6	2/6	0.5457	-0.6190	0.2188	0.3281	0.6065
IL-18	0 vs 10 cm	6	2/6	0.4703	-0.6190	0.2188	0.3281	0.6065
IL-18	2 vs 10 cm	6	3/6	1.4979	0.1429	0.8438	0.8438	0.6065
IL-1β	0 vs 2 cm	6	3/6	0.8145	-0.4286	0.4375	0.4375	0.2707
IL-1β	0 vs 10 cm	6	4/6	1.8241	0.5238	0.3125	0.4375	0.2707
IL-1β	2 vs 10 cm	6	6/6	1.7261	1	0.0312	0.0938	0.2707

Table S2A. Cross-cohort synthesis using Fisher's combined probability method.

Fisher's method combines cohort-specific p-values without pooling patient-level measurements. The 2-cm versus 10-cm contrast was not prespecified before examination of the data and is interpreted as exploratory and hypothesis-generating. q4 denotes Benjamini–Hochberg correction across the four proteins within a given spatial contrast, whereas q12 denotes the comprehensive correction across all 12 protein × contrast combinations. Fisher results are interpreted directionally only when cohort-specific effects are concordant.

Protein	Contrast	Direction concordant	Fisher p	BH q4	BH q12
NLRP3	0 vs 2 cm	Yes	0.2550	0.2956	0.3825
NLRP3	0 vs 10 cm	No	0.0660	0.0880	0.1584
NLRP3	2 vs 10 cm	No	0.7438	0.7438	0.7438
Caspase-1	0 vs 2 cm	No	0.2290	0.2956	0.3825
Caspase-1	0 vs 10 cm	No	0.0171	0.0457	0.0913
Caspase-1	2 vs 10 cm	Yes	0.4745	0.6327	0.5695
IL-18	0 vs 2 cm	No	0.0492	0.1970	0.1477
IL-18	0 vs 10 cm	No	0.0228	0.0457	0.0913
IL-18	2 vs 10 cm	Yes	0.2452	0.4903	0.3825
IL-1β	0 vs 2 cm	Yes	0.2956	0.2956	0.3942
IL-1β	0 vs 10 cm	No	0.6556	0.6556	0.7152
IL-1β	2 vs 10 cm	Yes	0.00421	0.0168	0.0505

Table S2B. Between-cohort heterogeneity of topographic contrast magnitude.

Exact Mann–Whitney tests compare patient-level log2 expression ratios between treatment-status cohorts. q4 denotes Benjamini–Hochberg correction across the four proteins within a given spatial contrast, and q12 denotes the comprehensive correction across all 12 protein × contrast combinations. Between-cohort heterogeneity should not be interpreted as a treatment effect because treatment status was confounded with baseline disease severity.

Protein	Contrast	Heterogeneity p	BH q4	BH q12
NLRP3	0 vs 2 cm	0.1812	0.2415	0.3105
NLRP3	0 vs 10 cm	0.0293	0.1083	0.1172
NLRP3	2 vs 10 cm	0.5728	0.8518	0.7637
Caspase-1	0 vs 2 cm	0.0293	0.1172	0.1172
Caspase-1	0 vs 10 cm	0.0593	0.1083	0.1625
Caspase-1	2 vs 10 cm	0.6620	0.8518	0.7944
IL-18	0 vs 2 cm	0.0813	0.1625	0.1625
IL-18	0 vs 10 cm	0.0813	0.1083	0.1625
IL-18	2 vs 10 cm	0.8518	0.8518	0.9293
IL-1β	0 vs 2 cm	1	1	1
IL-1β	0 vs 10 cm	0.2284	0.2284	0.3427
IL-1β	2 vs 10 cm	0.00799	0.0320	0.0959

Table S2C. Pooled and sign-test sensitivity analyses.

The pooled Wilcoxon analysis treats all 14 HS patients as one paired sample and is retained only as a supportive sensitivity analysis. The exact sign test evaluates directional consistency without using effect magnitude. q4 denotes Benjamini–Hochberg correction across the four proteins within a given spatial contrast, whereas q12 denotes the comprehensive correction across all 12 protein × contrast combinations. These pooled analyses do not replace the cohort-specific or primary mixed-design analyses.

Protein	Contrast	Pooled p	BH q4	BH q12	A>B / n	Sign p	Sign q4	Sign q12
NLRP3	0 vs 2 cm	0.1040	0.3875	0.5558	4/14	0.1796	0.4240	0.5387
NLRP3	0 vs 10 cm	0.3258	0.4631	0.5558	7/14	1	1	1
NLRP3	2 vs 10 cm	1	1	1	9/14	0.4240	0.5653	0.5653
Caspase-1	0 vs 2 cm	0.3575	0.4767	0.5558	5/14	0.4240	0.4240	0.5653
Caspase-1	0 vs 10 cm	0.3910	0.4631	0.5558	10/14	0.1796	0.7183	0.5387
Caspase-1	2 vs 10 cm	0.2412	0.3216	0.5558	8/14	0.7905	0.7905	0.8624
IL-18	0 vs 2 cm	0.6257	0.6257	0.6826	9/14	0.4240	0.4240	0.5653
IL-18	0 vs 10 cm	0.4631	0.4631	0.5558	9/14	0.4240	0.8479	0.5653
IL-18	2 vs 10 cm	0.1937	0.3216	0.5558	10/14	0.1796	0.3591	0.5387
IL-1β	0 vs 2 cm	0.1937	0.3875	0.5558	5/14	0.4240	0.4240	0.5653
IL-1β	0 vs 10 cm	0.4263	0.4631	0.5558	8/14	0.7905	1	0.8624
IL-1β	2 vs 10 cm	0.000244	0.000977	0.00293	13/14	0.00183	0.00732	0.0220

Table S3A. Partial F tests for the contribution of Western blot gel to between-patient expression variability.

For each protein, patient-level mean log2 expression across 0/2/10 cm was modeled using treatment-status cohort and gel as categorical predictors. The partial F test compares the full model (cohort + gel) with a reduced model containing cohort alone. F is the partial F statistic; df1 is the numerator degrees of freedom corresponding to the jointly tested gel terms, and df2 is the residual denominator degrees of freedom. BH q-values are adjusted across the four proteins.

Protein	Partial F statistic	df1	df2	p	BH q
NLRP3	0.6941	2	10	0.5221	0.6961
Caspase-1	1.2648	2	10	0.3238	0.6961
IL-18	0.1002	2	10	0.9056	0.9056
IL-1β	0.7481	2	10	0.4980	0.6961

Table S3B. Exact between-gel comparisons within available strata.

Exact permutation tests compare subject-level mean log2 expression between gels within strata represented on more than one gel. These analyses supplement the model-based gel assessment and do not by themselves establish absence of smaller batch effects.

Protein	Stratum	Gel comparison	n A	n B	Geometric ratio B/A	Exact p
NLRP3	Control	1 vs 2	3	2	0.2628	0.2000
NLRP3	HS_untreated	1 vs 2	6	2	1.0326	0.8214
NLRP3	HS_adalimumab	2 vs 3	4	2	0.6493	0.4000
Caspase-1	Control	1 vs 2	3	2	0.5004	0.5000
Caspase-1	HS_untreated	1 vs 2	6	2	0.8225	0.3571
Caspase-1	HS_adalimumab	2 vs 3	4	2	1.4990	0.3333
IL-18	Control	1 vs 2	3	2	0.3293	0.1000
IL-18	HS_untreated	1 vs 2	6	2	1.2285	0.4286
IL-18	HS_adalimumab	2 vs 3	2	4	0.9082	0.9333
IL-1β	Control	1 vs 2	3	2	0.5143	0.5000
IL-1β	HS_untreated	1 vs 2	6	2	0.8912	0.7143
IL-1β	HS_adalimumab	2 vs 3	4	2	1.5476	0.4000

Table S3C. Sensitivity analysis of the 2-cm/10-cm within-patient contrast across gels.

Exact Mann–Whitney tests compare patient-level log2(2 cm/10 cm) contrasts between gels within each treatment-status cohort.

Protein	Cohort	Gel comparison	n A	n B	Exact p	Ratio-scale shift
NLRP3	HS_untreated	Gel 1 vs Gel 2	6	2	0.1429	1.3910
NLRP3	HS_adalimumab	Gel 2 vs Gel 3	4	2	0.2667	9.0495
Caspase-1	HS_untreated	Gel 1 vs Gel 2	6	2	0.6429	0.6834
Caspase-1	HS_adalimumab	Gel 2 vs Gel 3	4	2	0.8000	0.6624
IL-18	HS_untreated	Gel 1 vs Gel 2	6	2	1	1.2485
IL-18	HS_adalimumab	Gel 2 vs Gel 3	2	4	0.5333	1.9670
IL-1β	HS_untreated	Gel 1 vs Gel 2	6	2	0.4286	1.1245
IL-1β	HS_adalimumab	Gel 2 vs Gel 3	4	2	0.5333	1.4812

Table S4A. Secondary all-gel comparisons with healthy controls.

Healthy controls, biologic-untreated HS, and adalimumab-treated HS were compared separately at each HS biopsy site using the Kruskal-Wallis test. BH q-values are adjusted across all 12 protein × site comparisons.

Protein	Site (cm)	Kruskal–Wallis H	p	BH q
NLRP3	0	2.7411	0.2540	0.4354
NLRP3	2	1.2063	0.5471	0.6565
NLRP3	10	3.7450	0.1537	0.3249
Caspase-1	0	6.9345	0.0312	0.1872
Caspase-1	2	2.3200	0.3135	0.4559
Caspase-1	10	0.1524	0.9266	0.9266
IL-18	0	2.1463	0.3419	0.4559
IL-18	2	3.7168	0.1559	0.3249
IL-18	10	3.6345	0.1625	0.3249
IL-1β	0	3.8842	0.1434	0.3249
IL-1β	2	0.3316	0.8472	0.9242
IL-1β	10	7.2292	0.0269	0.1872

Table S4B. Same-gel sensitivity analysis restricted to Gel 2.

Exact permutation-based three-group comparisons were performed using only samples measured on Gel 2. BH q-values are adjusted across the 12 protein × site tests.

Protein	Site (cm)	Exact H	Exact p	BH q	Permutations
NLRP3	0	1.8333	0.4857	0.8714	420
NLRP3	2	4.5000	0.0905	0.5429	420
NLRP3	10	6	0.0143	0.1714	420
Caspase-1	0	1.7917	0.5143	0.8714	420
Caspase-1	2	1.3333	0.5810	0.8714	420
Caspase-1	10	0	1	1	420
IL-18	0	3.4286	0.3333	0.8714	90
IL-18	2	0.2857	0.9333	1	90
IL-18	10	2	0.5333	0.8714	90
IL-1β	0	0	1	1	420
IL-1β	2	1	0.6952	0.9270	420
IL-1β	10	4.1250	0.1524	0.6095	420
